# Protocol for the development of a reporting guideline for clinical trials with integrated Chinese and western medicine interventions: the CONSORT extension for ICWM

**DOI:** 10.3389/fmed.2023.1190560

**Published:** 2023-06-29

**Authors:** Juan Wang, Xuan Zhang, Ping Wang, Fei Han, Ji Li, Yanfang Ma, Aiping Lyu, Zhaoxiang Bian

**Affiliations:** ^1^Chinese EQUATOR Centre, Chinese Clinical Trial Registry (Hong Kong), Hong Kong Chinese Medicine Clinical Study Centre, School of Chinese Medicine, Hong Kong Baptist University, Kowloon, Hong Kong SAR, China; ^2^Centre for Chinese Herbal Medicine Drug Development, Hong Kong Baptist University, Kowloon, Hong Kong SAR, China; ^3^Xiyuan Hospital, China Academy of Chinese Medical Sciences, Beijing, China; ^4^Department of Pediatrics, Guang’anmen Hospital, China Academy of Chinese Medical Sciences, Beijing, China

**Keywords:** CONSORT, ICWM, reporting guideline, Delphi survey, Chinese medicine

## Abstract

**Background:**

While Integrated Chinese and Western Medicine (ICWM) has become widely accepted as a necessary intervention for treating various diseases, key information about ICWM interventions is often missing in published clinical trials. To facilitate complete, transparent, and consistent reporting of clinical trials with ICWM interventions, an extension of the CONSORT guideline is necessary to be developed: the CONSORT-ICWM guideline.

**Methods:**

The CONSORT-ICWM guideline will be developed in five stages in accordance with recommendations for the development of reporting guidelines from the EQUATOR (Enhancing the QUAlity and Transparency Of health Research) Network, including (1) project launch and registration; (2) literature review and checklist draft; (3) Delphi survey; (4) consensus meeting; and (5) finalization of the guideline. Additionally, the working group will be composed of professors with expertise in integrated medicines, traditional Chinese medicines, biomedical informatics, statistics, methodology, development of reporting guidelines, epidemiology, health economics, and paper publications.

**Discussion:**

The CONSORT-ICWM guideline is to improve the reporting quality of clinical trials with ICWM interventions by ensuring the reports are complete, informative, clear, and transparent.

## Background

In recent years, Integrated Chinese and Western Medicine (ICWM) have been widely practised in the clinic, which is a treatment by Western medicine (WM) in conjunction with traditional Chinese medicine (TCM). TCM originated in China 5,000 years ago, following a unique and complete theoretical framework and using its terminology of etiology and pathogenesis. According to National Center for Complementary and Alternative Medicine (NCCAM), TCM comprises a variety of practices, including Chinese pharmacologic medicine (herbal, animal and mineral medicine), acupuncture, moxibustion (burning a herb above the skin to apply heat to acupuncture points), tuina (Chinese therapeutic massage), dietary therapy, and Daoyin therapy (such as baduanjin, tai chi and Qigong, which are practices that combine specific movements or postures, coordinated breathing, and mental focus) (1, 2). WM is several hundred years old but has rapidly progressed due to the advanced sciences and superb technologies. The WM is widely accepted and applied relying on robust evidence from both clinical research and evidence-based medicine (identifying a question, searching for evidence, appraising the evidence, applying the evidence, and assessing the evidence), which leads to evidence-based clinical practice guidelines (3). Based on a conclusive theoretical background, Chinese and Western medicine have followed various routines to gain knowledge in diagnosing and treating specially defined diseases, leading to reproducible treatment results (1). WM and TCM follow various routines to gain knowledge in diagnosing and treating specially defined diseases. The last centuries have brought about a transformation; modern Western medical procedures now frequently confirm empirical knowledge from the East. Recently, TCM has been used as adjuvant therapy in WM for patients with specific diseases, such as hypertension, coronary heart disease, pharyngitis, stroke, diabetes, and pediatric tic disorder, and they are enhancing the therapeutic effect without increasing adverse effects (4–10).

The COVID-19 pandemic has brought the ICWM of fresh evidence of efficacy and safety into stark relief, as multiple Chinese medical interventions may be beneficial to improve the symptoms and outcomes of patients (11, 12). Non-pharmacologic therapies in TCM, such as acupuncture, tuina, moxibustion, Qigong, and Daoyin therapy, combined with modern WM, have positive effects on multiple disorders (13–16). Otherwise, a systematic review demonstrated that ICWM could relieve some symptoms, such as fever, cough, expectoration, fatigue, chest tightness and anorexia; but the quality of the randomized controlled trials included in this systematic review was low, which resulted in the low confidence and feasibility of the evidence (17).

While many systematic reviews of ICWM reported some benefits from this combination, and there are many randomized controlled trials of ICWM compared to WM and showed that ICWM has a better therapeutic effect, no approbation and application of ICWM was provided for clinical practices widely all over the world. For example, Mohammed et al. conducted a systematic review and meta-analysis which included 29 studies to evaluate the effectiveness and safety of ICWM in comparison with WM in reducing systolic and diastolic blood pressure for patients with primary hypertension and the findings were supportive for ICWM interventions (5). A randomized, double-blind, and placebo-controlled trial has been conducted, and its findings proved that Xiaoke Pill, which is an ICWM formula composed of glibenclamide and several herb components from seven Chinese herbal medicines, led to evidential improvements in glycemic control and a significant reduction in risk of hypoglycemia after 48 weeks compared with glibenclamide (18, 19). But the ICWM interventions were merely recommended as a therapy in the clinical practice guidelines for treating hypertension or diabetes. Other than simplex TCM or WM interventions, it combines two different systematic ideologies and methodologies in diagnosis, treatment, outcomes measures, and prognosis, both from TCM and WM, which makes it difficult to assess the efficacy and safety of ICWM. The indicators of diagnosis and outcome mainly locate in symptoms and syndromes in TCM, but in changes of physiological markers in WM, so it is more complicated to evaluate the efficacy of ICWM interventions. At the same time, lacking tools that could evaluate the outcomes objectively, quantitatively, normatively, and practicably in clinical trials hinders the entire presentation of efficacy and safety of ICWM interventions, so that the trials and findings are not ratified by most researchers and clinicians.

Key information about ICWM interventions is often missing in published clinical trials. For instance, a previous study evaluating the reporting and quality of 78 clinical trials involving ICWM interventions in treating cough variant asthma found that the allocation, concealment and mechanism of randomization were lacking in all reports, the eligibility criteria for participants were missing in more than half of the reports, and there were large inaccuracy and variability in reporting of appraisal process used for outcome measurement properties (20). Another study evaluating the quality of 103 clinical studies on randomized controlled trials of Chinese herbal medicine combined with chemotherapy for hepatocellular carcinoma only covered 70% of items in the CONSORT statement in the 103 trial reports. Moreover, in most reports, the methods for statistics and randomization were not clearly described (over 95%), or the accuracy of harmful effects of Chinese herb medicine was not reported at all (84%) (21). Lacking key information in published reports hinders the appraisal of the efficacy and safety of ICWM interventions, and it implies the debatable quality of the design and conduction of the ICWM clinical trials. This situation might affect the determination of knowledge users (e.g., reviewers, healthcare providers, policymakers, regulators and patients) regarding the appropriateness of clinical trials involving ICWM interventions. Therefore, there is a need to develop a guideline in regard to the reporting of clinical trials involving ICWM interventions.

Reporting guidelines and standards are developed to facilitate and improve the completeness, clarity and transparency of different types of studies, trials, and outcomes. With respect to randomized trials, the Consolidated Standards of Reporting Trials (CONSORT) 2010 statement and its extensions are best known for providing reporting guidance (22). Moreover, the CONSORT statement captures some key sections that are also included in the reports of randomized trials involving the interventions of ICWM, such as trial design, participants, interventions, outcomes, randomization, and blinding (22). Actually, the quality of randomized controlled trials has taken a significant improvement since the CONSORT statement was published, which indicated that the standard reporting played a positive role in the design and conduction of clinical studies (23). Consequently, this protocol outlines the development and implementation process for a comprehensive and informative reporting guideline for clinical trials involving ICWM interventions extending from the CONSORT statement—the CONOSRT-ICWM guideline. The CONSORT-ICWM guideline will replenish crucial information necessary and specific to ICWM interventions, such as the necessity of the combinations of TCM and WM, how to combine the TCM and western medical interventions, when to perform the ICWM interventions, and so on. This information is required to make clinical trials with ICWM interventions reproduced and interpretable, which may contribute to the improvement of evidence and trustworthiness in all communities for proper use. Through an evidence-based and consensus-based process, the CONSORT-ICWM extension will evaluate what constitutes to crucial reporting of clinical trials employing ICWM interventions.

## Methods

**Figure 1:**
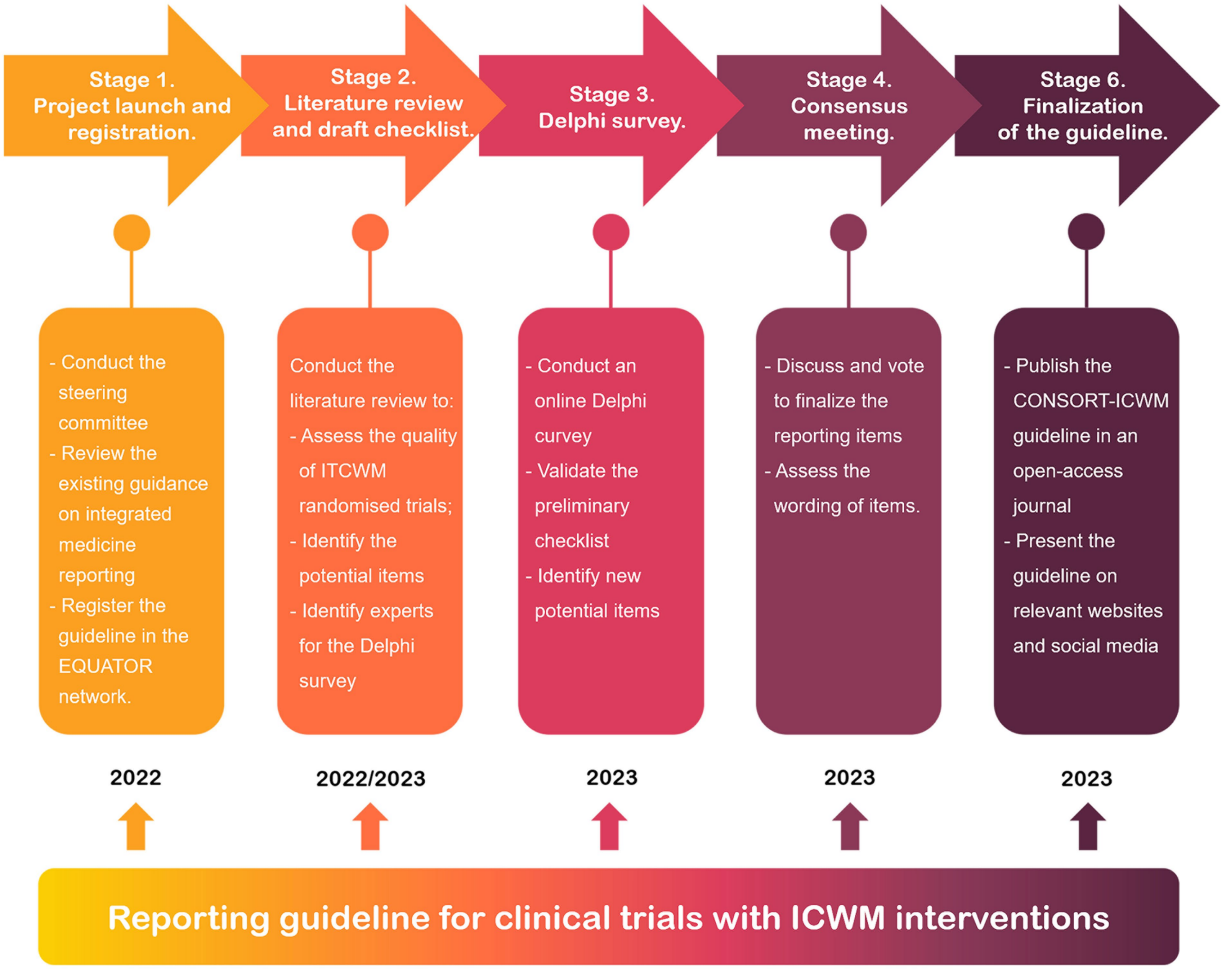
The outline of the development of the CONSORT-ICWM guideline.

The CONSORT-ICWM guideline will be developed in accordance with recommendations for reporting guideline development from the Enhancing the QUAlity and Transparency Of health Research (EQUATOR) Network (24). The development process for the CONSORT-ICWM guideline consists of the following five stages, depicted in Figure 1: (1) project launch and registration; (2) literature review and checklist draft; (3) Delphi survey; (4) consensus meeting; and (5) finalization of the guideline.

### Stage 1. Project launch and registration

The CONSORT-ICWM will be supported by the Chinese EQUATOR centre that seeks to improve the reliability and value of published health research literature by promoting transparent and accurate reporting and wider use of robust reporting guidelines (25). Director of the Chinese EQUATOR Centre, Professor Bian, will indicate and administrate this project, who have participated (as main author) and finished the development of CONSORT extensions for Chinese herbal formulas, moxibustion, and cupping. The experienced researchers with collective international expertise in reporting integrated medicine, guideline development, and knowledge synthesis will be invited as members of the steering committee (as co-authors in the finalized manuscript). Meanwhile, the CONSORT-ICWM will be registered on the EQUATOR Network library.

Additionally, the working group will be expanded with external advisors with expertise in integrated medicines, TCM, WM, biomedical informatics, statistics, methodology, development of reporting guidelines, conduction of clinical trials, epidemiology, health economics, and paper publications. The external advisors will be screened through the attendees of related conferences, editors of associated books or journals, experts identified by the committee, and authors of related studies. The expanded work group will make its efforts to assure diversity and variety in research areas, career stages, geography, gender, and multicultural representation. We plan to invite researchers, practitioners, and candidates coming from China Mainland, Hong Kong, Tai Wan, Japan, Canada, the United States, the United Kingdom, Australia, Italy, and South Korea. Due to their large and international network, other panelists, pilot testers, as well as consensus meeting conventioners will be identified to participate in this project as external advisors. All the participants will be appreciated in the section of acknowledgement in the finalized manuscript. The responsibilities of each work group were described in Table 1.

**Table 1:** Responsibilities of work groups in the development of CONSORT-ICWM guideline.

Work groups	Responsibility
Steering committee	(1) Lead the study and provide oversight for the project; (2) Provide all the necessary materials and methods for each stage; (3) Prepare the documentation for the institutional review board; (4) Register to EQUATOR Network; and (5) Identify and invite experts for the Delphi survey and consensus meeting.
Expanded work group	(1) Review existing CONSORT/SPIRIT reporting guidelines related to TCM interventions and integrated interventions; (2) Evaluate the published scoping reviews and clinical trials related to ICWM; (3) Generate the candidate items of the checklist; (4) Design the international Delphi survey; (5) Organize the consensus meeting; (6) Develope the final CONSORT-ICWM guideline; and (7) Write the manuscripts and submitting the final products to peer-review, publication, and dissemination.
External advisors	(1) Participate in Delphi survey; (2) Attend the consensus meeting; (3) Participate in the polit test; and (4) Comment or suggest for the CONSORT-ICWM guideline during /after the project.

### Stage 2. Literature review and checklist draft

The purpose of the literature review is threefold: (1) to build a knowledge base on the quality of reporting of the clinical trials involving ICWM interventions, including the parts of study design, methodological methods, results, harms of TCM and WM, and related interests; (2) to provide insight into possible reporting items for CONSORT-ICWM guideline; and (3) to suggest a list of advisors who have published or editing the ICWM clinical research as potential participants for the Delphi survey.

The literature review will be developed following the recommendation of the Preferred Reporting Items for Systematic reviews and Meta-Analyses (PRISMA) 2020 and PRISMA-Search extension guidelines (26, 27). And searches of Embase (Ovid), AMED (Ovid), Cochrane central register f controlled trials (Ovid), Medline (Ovid), PubMed, CNKI, and WANFANG databases will be run with no limits by year or language of publications (search strategy in Additional File 1). The primary search strategy will be developed and tested by members of the steering committee with the assistance of informatics and evidence-based medical specialists. The testing searches allow the adjustments of the primary search strategy to produce literature that is better aligned with this project’s inclusion criteria and aim. We will adjust the search strategy for every database to obtain refined and broad results. The additional relevant articles will be collected by the members of the steering committee based on the individuals’ prior work and expertise in the area via a manual search.

The CONSORT-ICWM guideline will serve various types of clinical trials, including randomized trials, observational studies, case reports, and diagnostic/prognostic studies. So apart from the literature of ICWM trials, we will review several relevant reporting guidelines to develop the recommendations for ICWM clinical trials, such as CONSORT extensions for randomized trials, STROBE (The Strengthening the Reporting of Observational Studies in Epidemiology) statement and extensions for observational studies, SPIRIT (Standard Protocol Items: Recommendations for Interventional Trials) statement and extensions for protocol of clinical trials, CARE (CAse Report) guideline and extensions, STARD (the Standards for Reporting of Diagnostic Accuracy Studies) statements for diagnostic studies, and TRIPOD (Transparent Reporting of a multivariable prediction model for Individual Prognosis Or Diagnosis) statement for prognostic and diagnostic studies (22, 28–32).

Using the CONSORT 2010 statement as a starting point, new items will be refined by reviewing the clinical trials with ICWM interventions and the extensions for Chinese herbal formulas, acupuncture and moxibustion. A table detailing all possible additional reporting items and initial reporting items from the CONSORT statement will be presented to the entire steering committee. After the expert consultations, the preliminary CONSORT-ICWM checklist will be drafted and modified based on the feedback from the steering committee.

### Stage 3. Delphi survey

The steering committee and technical advisory group will identify all relevant organizations, councils, groups, and individuals through their professional networks and affiliations. Maximum variation with respect to the professional background and work experience of panelists will be sought. Known panelists from other relevant reporting guidelines, researchers or clinicians of ICWM, and authors or editors who have conducted clinical trials with ICWM interventions will be invited. Patients/potential users in complementary and alternative medicine (CAM) or WM will be recruited through social media channels, university websites, and contact persons of relevant organizations. There will be no geographical restrictions on eligibility. We will send the first-round invitations to 120 candidate participants who come from all around the world and at most five persons in one region. The invitation lists will be expanded to guarantee the diversity of background, work experience, and geography of the panelists according to the responses during the Delphi survey.

Every candidate item in the Delphi questionnaires will be shown in English, simplified Chinese and traditional Chinese at the same time. Panelists will be sent email-based invitations, including the objective and introduction of this project, explanations of the Delphi exercise, as well as the informed consent of comments collection and confidentiality of responses during the Delphi process. A minimum of 30 panelists will be considered appropriate (33, 34). According to previous experiences, it is anticipated that, at the most 50% of the invited persons will complete at least one round. Therefore, no less than 60 people should be willing to complete round 1 (35, 36). Maximum variation with respect to the professional background and work experience of panelists will be sought.

The preliminary checklist agreed on by the steering committee will subsequently be put to the CONSORT-ICWM Delphi Panel for validation with an online platform. The online Delphi questionnaire will be developed and maintained through the QuestionPro Survey Software (Hong Kong Baptist University). The Delphi survey will be conducted to review and refine the CONSORT-ICWM reporting items and make the best use of all available information. Based on the candidate items generated in Stage 2, a list of reporting items, together with the operational definitions and examples, will be developed for use in the Delphi survey. The entire questionnaire for the first round of the Delphi survey, consisting of invitation texts, will be designed and pilot examined with the steering committee and technical advisory group. Feedback on the layout will be accumulated, and the questionnaire will be revised accordingly. The Guidance on conducting and REporting DElphi Studies (CREDES), specific to the palliative care, will be applied for reviewing the elements that can be verbalized to other biomedical research as well (37).

An anonymous online survey will be conducted where panelists can evaluate each candidate item in relation to its importance and relevance for the CONSORT-ICWM guideline, using a five-point scale, together with a free text box for comments. Panelists will be asked to rate each item on a 5-point scale (1-definitely reject, 2-probably reject, 3-neutral, 4-probably keep, 5-definitely keep). After the checklist, there will be the option of “other recommendation or reason” for the experts or the patients/potential users to collect expanded suggestions for the project. Panelists will be encouraged to provide a rationale for their ratings, to indicate modifications of definitions or words of items, and to suggest new items that are not included in the checklist. Following each round, the score for each item will be calculated with the formula of 100% * (1*N5 + 0.75*N4 + 0.5*N3 + 0.25*N2)/(N5 + N4 + N3 + N2 + N1), where Ni represents the number of respondents who chose specific “i” in the scale of “1 to 5.” Items with a score greater than or equal to 75% will be kept. In this formula, both the consensus level and the weight of responses will be considered (38). Any items that do not reach consensus or any new items, together with a comment summary of each item, will be circulated in subsequent rounds.

Proposals for modifications, aggregation or new items will be considered and deliberated by the steering committee and integrated into following round. All results will be summarized into a feedback report that will be sent to the panelists with email along with the online survey link and QR code of the following round. The Delphi survey will be closed after the required sample is reached, or a maximum of 8 weeks will be provided for responding to the survey. Three times of reminders prior to the round’s closing date will be sent to the panelists in order to minimize the non-response bias. And anonymity and confidentiality of responses will be ensured.

### Stage 4. Consensus meeting

On completion of the Delphi survey, a consensus meeting will be organized online via the Tencent meeting platform to review the results and obtain the expert consensus on which items will be contained, along with their finalized wording in the CONSORT-ICWM guideline. Besides the steering committee, experts in trial methodology and reporting guideline development, clinical practice of Chinese and Western medicine, epidemiology, and medical journal editing will be invited to attend the consensus meeting. Besides, the experts who have participated in the Delphi survey will not be invited to the consensus meeting.

A separate explanation and elaboration (E&E) document will be created, which will provide the background, rationale, justification, and examples from good reporting for each item included in the reporting guideline. Each candidate item will be presented in a consensus meeting to all professionals, together with the Delphi Questionnaires, results from the three-round Delphi survey, and draft E&E documents. All participants will discuss and refine each item in the checklist, after which anonymous voting will be conducted to decide the inclusion and precise wording of each item. Consensus for exclusion or inclusion of an item will be reached if at least 70% of the participants vote for exclusion/inclusion. The round table discussion and vote process will be repeated for any issues that do not receive unanimous support. The round table discussion and vote process will be repeated for any items that do not reach a consensus. This process will continue until all items have reached a consensus. At the end of the meeting, we will present the data results to participants to further confirm that their recommendations are appropriately understood and considered, after which the experts should check and sign the final guideline.

### Stage 5. Finalization of the guideline

We intend to publish the CONSORT-ICWM extensions and E&E document in a peer-review journal. Throughout the project, the active presence of the CONSORT-ICWM guideline on relevant websites (e.g., the EQUATOR website) and social media (e.g., Facebook, Twitter, and LinkedIn) will be maintained. We will report the project and the CONSORT-ICWM guideline at relevant conferences. In addition, the CONSORT-ICWM and E&E documents will be published in open-access journals, and readers can get them through relevant websites, such as the CONSORT website,^1^ the EQUATOR website,^2^ and the Chinese EQUATOR centre website.^3^ Other strategies for dissemination of the reporting guideline will be considered in practice.

## Discussion

The CONSORT-ICWM guideline will provide a set of minimum items on what should be reported in clinical trials involving ICWM interventions. The CONSORT-ICWM guideline will be created with the intention of harmonizing and standardizing the reporting of clinical trials, which can facilitate making the ICWM clinical trials reproducible and transparent, and reduce research wastes.

The evidence-based and consensus-based methods that will be conducted in the process of development will contribute to the acceptance and assimilation of the CONSORT-ICWM guideline by organizations, journals, and individual users. With the engagement of a large group of experts from different scientific domains and international organizations representing multiple perspectives, the CONSORT-ICWM guideline has a good prospect of becoming widely used in practice. Roundtable discussion and negotiation will be conducted when divarication exists, and the steering committee will decide on the consensus rules.

The ICWM interventions will be different in treating distinct disorders, especially pediatric diseases. Therefore, based on the CONSORT-ICWM guideline, the workgroup will develop two extended reporting guidelines for clinical trials with ICWM interventions involving precancerous lesions of gastric cancer and pediatric tic disorder, to direct future users who need to employ the CONSORT-ICWM guideline in a specific disease. The execution and evaluation of the CONSORT-ICWM guideline will lead to the identification of barriers and facilitators, and lessons learned. The practitioners, trialists, and researchers who are relevant to precancerous lesions of gastric cancer and pediatric tic disorder will be, respectively, invited to participate in the development of the extended reporting guidelines as well. We will develop the extended guidelines following the methodology and process recommended by the EQUATOR network, including prospective registration, literature review, Delphi survey, consensus meeting, and finalization and dissemination of the guidelines. The detailed procedure and findings will be presented and shared in open-access platforms and journals.

There are some limitations to this work. First, the specific ways of combining TCM and WM are various, such as overlying, one-after-another, and add-on design, and thus the guideline could present only some detailed items related to one specific type. Hence, we will try our best to enhance the diversity of background, work experience, and geography of the work groups to reduce the potential bias and increase the external validation. Second, although the development of the CONSORT-ICWM extension is based on a standard methodology process, we continually welcome additional expressions of interest and suggestions for relevant literature. We plan to engage in this work to ensure meaningful engagement and enhance the practicality.

## Conclusion

We will use an iterative process to draft, refine, and finalize the guideline to be provided in each manuscript in consultation with the steering committee. The expected final guideline will be propitious to improve the interpretability and reproducibility of clinical trials involving ICWM interventions, ensuring that the reports are complete, informative, and transparent, thus enhancing the ICWM interventions applied standardizedly and widely in the clinic.

## Ethics statement

Since this research does not use health data of individuals, ethics approval is not required according to the Ethics Committee of Hong Kong Baptist University, Hong Kong. The survey and meeting participants will be required to provide their consent prior to participating in the survey.

## Author contributions

ZB and XZ concepted and designed the project. JW and XZ drafted and revised the manuscript. FH, JL, and YM provided critical comments for the manuscript. ZB, AL, and XZ provided the administrative, technical, or logistic supports. All authors read and approved the final manuscript.

## Funding

This work was supported by the China Center for Evidence Based Traditional Chinese Medicine, CCEBTM (2020YJSZX-5). The funders had no role in the design of the study, in the collection, analysis, and interpretation of data, nor in the writing of the manuscript.

## Conflict of interest

The authors declare that the research was conducted in the absence of any commercial or financial relationships that could be construed as a potential conflict of interest.

## Publisher’s note

All claims expressed in this article are solely those of the authors and do not necessarily represent those of their affiliated organizations, or those of the publisher, the editors and the reviewers. Any product that may be evaluated in this article, or claim that may be made by its manufacturer, is not guaranteed or endorsed by the publisher.

## Footnotes

^1^https://www.consort-statement.org/

^2^https://www.equator-network.org/

^3^http://www.equator-chinese.org/pages/cn/index/index.html

## Abbreviations

CAM: Complementary and alternative medicine
CONSORT: Consolidated Standards of Reporting Trials
CREDES: Guidance on conducting and REporting DElphi Studies
EQUATOR: Enhancing the QUAlity and Transparency of Health Research
E&E: Explanation and elaboration
ICWM: Integrated Chinese and Western Medicine
NCCAM: National Center for Complementary and Alternative Medicine
PRISMA: Preferred Reporting Items for Systematic Reviews and Meta-Analyses
TCM: Traditional Chinese medicine
WM: Western medicine
STROBE: The Strengthening the Reporting of Observational Studies in Epidemiology
SPIRIT: Standard Protocol Items: Recommendations for Interventional Trials
CARE: (CAse Report) guideline and extensions
STARD: the Standards for Reporting of Diagnostic Accuracy Studies
TRIPOD: Transparent Reporting of a multivariable prediction model for Individual Prognosis or Diagnosis

## References

[1] Lampe H Halle B Freund M. Western and Chinese medicine in oncology and hematology. Forsch Komplementmed. (2011) 18:185–91. doi: 10.1159/000330935 21934318

[2] National Center for Complementary and Alternative Medicine (NCCAM) traditional Chinese medicine: an introduction. Available at: http://nccam.nih.gov/health/whatiscam/chinesemed.htm (Accessed November 20, 2022).

[3] Szajewska H. Evidence-based medicine and clinical research: both are needed. Neither Perfect Ann Nutr Metab. (2018) 72:13–23. doi: 10.1159/000487375, PMID: 29631266

[4] Mohammed SAD Hanxing L Fang L Algradi AM Alradhi M Safi M . Integrated Chinese herbal medicine with Western medicine versus Western medicine in the effectiveness of primary hypertension treatment: a systematic review and meta-analysis of randomized controlled trials. J Ethnopharmacol. (2023) 300:115703. doi: 10.1016/j.jep.2022.115703, PMID: 36096347

[5] Hui J Yuan R Li P Xin Q Miao Y Shen X . Efficacy and safety of different courses of tongxinluo capsule as adjuvant therapy for coronary heart disease after percutaneous coronary intervention: a systematic review and meta-analysis of randomized controlled trials. J Clin Med. (2022) 11:2991. doi: 10.3390/jcm11112991, PMID: 35683377 PMC9181557

[6] Lyu J Yang C Wang LX Xie YM Yu XQ Gu L . Randomized double-blind parallel controlled multicenter trial of reyanning mixture in treatment of acute tonsillitis. Zhongguo Zhong Yao Za Zhi. (2020) 45:3282–91. doi: 10.19540/j.cnki.cjcmm.20200420.501 32726041

[7] Zhong LL Zheng Y Lau AY Wong N Yao L Wu X . Would integrated Western and traditional Chinese medicine have more benefits for stroke rehabilitation? A systematic review and meta-analysis. Stroke Vasc Neurol. (2022) 7:77–85. doi: 10.1136/svn-2020-000781, PMID: 34446530 PMC8899656

[8] Hu Y Zhou X Liu P Wang B Duan DM Guo DH. A comparison study of metformin only therapy and metformin combined with Chinese medicine jianyutangkang therapy in patients with type 2 diabetes: a randomized placebo-controlled double-blind study. Complement Ther Med. (2016) 24:13–8. doi: 10.1016/j.ctim.2015.11.005, PMID: 26860796

[9] Zhang LW Xie J. Meta-analysis of integrated traditional Chinese and western medicine treatment of tourette syndrome in children. Clin J Chinese Med. (2022) 14:20–4.

[10] Yan ZF Kong LB Wang SJ Liu BL Xu Y Liu YK . Clinical dominant disease in traditional Chinese medicine: a series of youth salon seminars for clinical dominant disease held by China association of Chinese medicine. Chin J Exp Tradit Med Formulae. (2023) 29:202–8. doi: 10.13422/j.cnki.syfjx.20230193

[11] Zhou S Feng J Xie Q Huang T Xu X Zhou D . Traditional Chinese medicine shenhuang granule in patients with severe/critical COVID-19: a randomized controlled multicenter trial. Phytomedicine. (2021) 89:153612. doi: 10.1016/j.phymed.2021.153612, PMID: 34126419 PMC8161732

[12] Hu K Guan WJ Bi Y Zhang W Li L Zhang B . Efficacy and safety of Lianhuaqingwen capsules, a repurposed Chinese herb, in patients with coronavirus disease 2019: a multicenter, prospective, randomized controlled trial. Phytomedicine. (2021) 85:153242. doi: 10.1016/j.phymed.2020.153242, PMID: 33867046 PMC7229744

[13] Wang T Xu C Pan K Xiong H. Acupuncture and moxibustion for chronic fatigue syndrome in traditional Chinese medicine: a systematic review and meta-analysis. BMC Complement Altern Med. (2017) 17:163. doi: 10.1186/s12906-017-1647-x, PMID: 28335756 PMC5363012

[14] Zhou M Zhang H Li F Yu Z Yuan C Oliver B . Pulmonary Daoyin as a traditional Chinese medicine rehabilitation programme for patients with IPF: a randomized controlled trial. Respirology. (2021) 26:360–9. doi: 10.1111/resp.13972, PMID: 33164264 PMC8048896

[15] Liu ST Zhan C Ma YJ Guo CY Chen W Fang XM . Effect of qigong exercise and acupressure rehabilitation program on pulmonary function and respiratory symptoms in patients hospitalized with severe COVID-19: a randomized controlled trial. Integr Med Res. (2021) 10:100796. doi: 10.1016/j.imr.2021.100796, PMID: 34733607 PMC8553411

[16] Lee NW Kim GH Heo I Kim KW Ha IH Lee JH . Chuna (or Tuina) manual therapy for musculoskeletal disorders: a systematic review and meta-analysis of randomized controlled trials. Evid Based Complement Alternat Med. (2017) 2017:8218139–22. doi: 10.1155/2017/8218139, PMID: 29441114 PMC5758860

[17] Liu M Gao Y Yuan Y Yang K Shi S Zhang J . Efficacy and safety of integrated traditional Chinese and Western medicine for Corona virus disease 2019 (COVID-19): a systematic review and meta-analysis. Pharmacol Res. (2020) 158:104896. doi: 10.1016/j.phrs.2020.104896, PMID: 32438037 PMC7211759

[18] Yeh GY Eisenberg DM Kaptchuk TJ Phillips RS. Systematic review of herbs and dietary supplements for glycemic control in diabetes. Diabetes Care. (2003) 26:1277–94. doi: 10.2337/diacare.26.4.1277, PMID: 12663610

[19] Ji L Tong X Wang H Tian H Zhou H Zhang L . Efficacy and safety of traditional Chinese medicine for diabetes: a double-blind, randomised, controlled trial. PLoS One. (2013) 8:e56703. doi: 10.1371/journal.pone.0056703, PMID: 23460810 PMC3584095

[20] Zhang S Zhang TH Li XJ Wang CJ Pan BF Zhang WW . Quality evaluation of literature of integrated Chinese-western therapy for cough varian asthma. Chin J Inform Tradit Chinese Med. (2012) 19:22–5.

[21] Meng MB Wang G Cui RL Yu B Zhang RM. Quality assessment of clinical studies on randomized controlled trials of Chinese herb medicine and chemotherapy in the treatment of hepatocellular carcinoma. West China Med J. (2008) 1:1–2.

[22] Moher D Hopewell S Schulz KF Montori V Gøtzsche PC Devereaux PJ . CONSORT 2010 explanation and elaboration: updated guidelines for reporting parallel group randomised trials. Int J Surg. (2012) 10:28–55. doi: 10.1016/j.ijsu.2011.10.001, PMID: 22036893

[23] Nayan M Jayalath VH Jewett MA Bedard PL Hamilton RJ. Randomized controlled trials in testicular cancer: a demographic and quality assessment. Urol Oncol. (2016) 34:60.e7. doi: 10.1016/j.urolonc.2015.09.007, PMID: 26493448

[24] Moher D Schulz KF Simera I Altman DG. Guidance for developers of health research reporting guidelines. PLoS Med. (2010) 7:e1000217. doi: 10.1371/journal.pmed.1000217, PMID: 20169112 PMC2821895

[25] Chinese EQUATOR Centre. Available at: https://www.equator-network.org/about-us/chinese-equator-centre/ (Accessed January 10, 2023).

[26] Page MJ McKenzie JE Bossuyt PM Boutron I Hoffmann TC Mulrow CD . The PRISMA 2020 statement: an updated guideline for reporting systematic reviews. BMJ. (2021) 372:n71. doi: 10.1136/bmj.n71, PMID: 33782057 PMC8005924

[27] Rethlefsen ML Kirtley S Waffenschmidt S Ayala AP Moher D Page MJ . PRISMA-S: an extension to the PRISMA statement for reporting literature searches in systematic reviews. Syst Rev. (2021) 10:39. doi: 10.1186/s13643-020-01542-z, PMID: 33499930 PMC7839230

[28] von Elm E Altman DG Egger M Pocock SJ Gøtzsche PC Vandenbroucke JP . The strengthening the reporting of observational studies in epidemiology (STROBE) statement: guidelines for reporting observational studies. Ann Intern Med. (2007) 147:573–7. doi: 10.7326/0003-4819-147-8-200710160-00010 17938396

[29] Chan AW Tetzlaff JM Altman DG Laupacis A Gøtzsche PC Krleža-Jerić K . SPIRIT 2013 statement: defining standard protocol items for clinical trials. Ann Intern Med. (2013) 158:200–7. doi: 10.7326/0003-4819-158-3-201302050-00583, PMID: 23295957 PMC5114123

[30] Gagnier JJ Kienle G Altman DG Moher D Sox H Riley D . The CARE guidelines: consensus-based clinical case reporting guideline development. BMJ Case Rep. (2013) 2013:bcr2013201554. doi: 10.1136/bcr-2013-201554, PMID: 24228906 PMC3844611

[31] Bossuyt PM Reitsma JB Bruns DE Gatsonis CA Glasziou PP Irwig L . STARD 2015: an updated list of essential items for reporting diagnostic accuracy studies. Clin Chem. (2015) 61:1446–52. doi: 10.1373/clinchem.2015.246280, PMID: 26510957

[32] Collins GS Reitsma JB Altman DG Moons KG. Transparent reporting of a multivariable prediction model for individual prognosis or diagnosis (TRIPOD): the TRIPOD statement. Ann Intern Med. (2015) 162:55–63. doi: 10.7326/M14-0697 25560714

[33] Akins RB Tolson H Cole BR. Stability of response characteristics of a Delphi panel: application of bootstrap data expansion. BMC Med Res Methodol. (2005) 5:37. doi: 10.1186/1471-2288-5-37, PMID: 16321161 PMC1318466

[34] Chalmers J Armour M. The Delphi technique In: Liamputtong P, editor. Handbook of research methods in health social sciences. Singapore: Springer (2019). 715–35.

[35] Cheng CW Wu TX Shang HC Li YP Altman DG Moher D . CONSORT extension for Chinese herbal medicine formulas 2017: recommendations, explanation, and elaboration. Ann Intern Med. (2017) 167:112–21. doi: 10.7326/M16-2977, PMID: 28654980

[36] MacPherson H Altman DG Hammerschlag R Li Y Wu T White A . Revised STandards for reporting interventions in clinical trials of acupuncture (STRICTA): extending the CONSORT statement. Acupunct Med. (2010) 28:83–93. doi: 10.1136/aim.2009.001370, PMID: 20615861 PMC3002761

[37] Jünger S Payne SA Brine J Radbruch L Brearley SG. Guidance on conducting and REporting DElphi studies (CREDES) in palliative care: recommendations based on a methodological systematic review. Palliat Med. (2017) 31:684–706. doi: 10.1177/0269216317690685, PMID: 28190381

[38] Chen Y Yang K Marušić A Qaseem A Meerpohl JJ Flottorp S . A reporting tool for practice guidelines in health care: the RIGHT statement. Ann Intern Med. (2017) 166:128–32. doi: 10.7326/M16-1565, PMID: 27893062

