# Supplementary material for: Protocol for the development of a reporting guideline for clinical trials with integrated Chinese and western medicine interventions: the CONSORT extension for ICWM
Source: Front Med (Lausanne). 2023 Jun 29;10:1190560. doi: 10.3389/fmed.2023.1190560 (PMC10344360; doi:10.3389/fmed.2023.1190560)
Supplement: Supplementary file 1 [file Presentation_1.pdf]

Additional file 1. Search strategies for literature review

The databases to be searched are:
PubMed; MEDLINE (OVID); Embase (OVID); AMED (OVID); Cochrane Central Register of Controlled Trials (OVID); CNKI; WANFANG.

Search strategies:
PubMed
https://pubmed.ncbi.nlm.nih.gov/advanced/
Published Date: 1946 to 30 November 2022
#1 Integrated Chinese and Western Medicine[All Fields] OR Integrative Medicine[All Fields] OR Combining Chinese and Western Medicine[All Fields] OR Combination of Chinese and Western Medicine[All Fields] OR Chinese and Western integrative medicine[All Fields] OR combined[Title/Abstract] OR combination[Title/Abstract])OR (integrated[Title/Abstract] OR based on controlled group[All Fields]
#2 Randomized controlled trial[MeSH Terms] OR Random allocation[MeSH Terms] OR (random*[Title/Abstract] AND (control*[Title/Abstract] OR placebo[Title/Abstract]))
#3 Medicine, Chinese Traditional[MeSH Terms] OR Drugs, Chinese Herbal[MeSH Terms] OR Traditional Chinese Medicine[Title/Abstract] OR Chinese medicine[Title/Abstract] OR herbal drug[All Fields] OR herbal medicine[All Fields] OR herb formula[All Fields]OR decoction[Title/Abstract] OR Chinese Medicine Patent Prescription[All Fields] OR proprietary Chinese medicines[All Fields] OR Chinese patent medicine[All Fields] OR (exp Acupuncture Therapy[Title/Abstract]) OR (exp Acupuncture[Title/Abstract]) OR (Electroacupuncture[Title/Abstract]) OR (Acupoint*[Title/Abstract]) OR (exp Massage/[Title/Abstract]) OR massage[Title/Abstract] OR tuina[Title/Abstract] OR Moxibustion/[Title/Abstract] OR moxa[Title/Abstract] OR moxibustion[Title/Abstract] OR meridian*[Title/Abstract] OR Acupoint application[Title/Abstract] OR Auricular acupoint[Title/Abstract] OR Cupping[Title/Abstract] OR Fumigation[Title/Abstract] OR (Guasha) OR (Daoyin or Qigong or taijiquan or Tai Chi or baduanjin[Title/Abstract]) OR wuxing[Title/Abstract]
#4 #1 AND #2 AND #3

Ovid (MEDLINE & Embase & AMED & Cochrane Central Register of Controlled Trials)
https://ovidsp.dc1.ovid.com/ovid-a/ovidweb.cgi
Published Date: EBM Reviews - Cochrane Central Register of Controlled Trials <30 November 2022>; AMED (Allied and Complementary Medicine) <1985 to 30 November 2022>; Embase <1974 to 30 November 2022>; Ovid MEDLINE(R) <1946 to 30 November 2022>.
#1 ((Integrated Chinese and Western Medicine) OR (Integrative Medicine) OR (Combining Chinese and Western Medicine) OR (Combination of Chinese and Western Medicine) OR (Chinese and Western integrative medicine)).mp. OR (combined:ti,ab,kw) OR (integrated:ti,ab,kw) OR (combination:ti,ab,kw) OR (based on controlled group.tw.)
#2 randomized controlled trial.mp. OR RCT:ti,ab,kw OR controlled clinical trial.mp. OR random$.tw. OR (crossover$ or cross over$ or cross-over$).kw. OR placebo$.tw. OR allocated$.tw.

#3 (exp Drugs, Chinese Herbal/ or Chinese Herbal Drugs.mp.) OR (Chinese Traditional Medicine.mp.) OR (Chinese medicine$ or traditional medicine$.mp.) OR (herbal drug$ or herbal medicine$.mp.) OR (herb$ or herb$ formula$ or decoction$.mp.) OR (Chinese Medicine Patent Prescription or proprietary Chinese medicines.mp.) OR (Chinese patent adj3 (medicine or drug$).mp.) OR (exp Acupuncture Therapy/) OR (exp Acupuncture/) OR (Electroacupuncture.tw,kf.) OR (acupuncture.tw,kf.) OR (Acupoint*.tw.) OR (exp Massage/) OR (massage* or tuina:ti,ab,kw) OR (Moxibustion/) OR ((moxa or moxibustion).mp.) OR (meridian*.tw,kf.) OR (Acupoint application.mp.) OR (Auricular acupoint.mp.) OR (Cupping.mp.) OR (Fumigation.mp.) OR (Guasha.mp.) OR (Daoyin or Qigong or taijiquan or baduanjin.mp.) OR (wuxing.mp.)
#4 #1 AND #2 AND #3

CNKI
https://kns.cnki.net/kns8/AdvSearch?dbprefix=CFLS&&crossDbcodes=CJFQ%2CCDMD%2CCIPD%2CCCND%2CCISD%2CSNAD%2CBDZK%2CCCJD%2CCCVD%2CCJFN
Published Date: 1998 to 30 November 2022.
TI=('临床试验'+'临床研究'+'随机'+'对照'+'随机试验'+'随机对照试验') AND SU=('中医'+'中医药'+'中药'+'复方'+'汤剂'+'中成药'+'注射剂 '+'加减方'+'针灸'+'针刺'+'穴位'+'贴敷'+'敷贴'+'耳穴'+'经络'+'灸'+'推拿'+'刮痧'+'拔罐'+'导引'+'太极'+'八段锦'+'气功'+'五行') AND SU=('中西医结合'+'中西结合'+'中西医联合'+'中西联用'+'中西医结合疗法'+'中西医结合试验'+'中西医结合治疗'+'中西医'+'中西') NOT TI = ('meta' + '系统评价' + '研究进展' + '研究现状' + '研究概况')

WANFANG
https://s.wanfangdata.com.cn/advanced-search/paper
Published Date: 1980 to 30 November 2022.
题名:(临床试验 or 临床研究 or 随机对照试验 or 随机 or 对照 or 随机试验 or 随机研究 or RCT) and 主题:(中医 or 中医药 or 中药 or 复方 or 汤剂 or 中成药 or 注射剂 or 加减方 or 针灸 or 针刺 or 穴位 or 贴敷 or 敷贴 or 耳穴 or 经络 or 灸 or 推拿 or 刮痧 or 拔罐 or 导引 or 太极 or 八段锦 or 气功 or 五行) and 主题:(中西医结合 or 中西结合 or 中西医联合 or 中西联用 or 中西医结合疗法 or 中西医结合试验 or 中西医结合治疗 or 中西医 or 中西) not (meta or 系统评价)

Additional file 2. Assessment of this protocol with RIGHT checklist.

RIGHT Checklist

RIGHT (Reporting Items for practice Guidelines in HealThcare) Checklist				
22 Sections/topics and 35 Items		Assessment	Page(s)	Note(s)
**Section/topic**	**No./ Item**			
**Basic information**				
**Title/subtitle**				
1a	Identify the report as a guideline, that is, with "guideline(s)" or "recommendation(s)" in the title.	☑ Yes ☐ No ☐ Unclear	1	
1b	Describe the year of publication of the guideline.	☐ Yes ☑ No ☐ Unclear	NA	
1c	Describe the focus of the guideline, such as screening, diagnosis, treatment, management, prevention, or others.	☑ Yes ☐ No ☐ Unclear	1	
**Executive summary**				

2	Provide a summary of the recommendations contained in the guideline.	☐ Yes<br>☑ No<br>☐ Unclear	NA	
**Abbreviations and acronyms**				
3	Define new or key terms, and provide a list of abbreviations and acronyms if applicable.	☐ Yes<br>☑ No<br>☐ Unclear	NA	
**Corresponding developer**				
4	Identify at least 1 corresponding developer or author who can be contacted about the guideline.	☑ Yes<br>☐ No<br>☐ Unclear	3	
**Background**				
**Brief description of the health problem(s)**				
5	Describe the basic epidemiology of the problem, such as the prevalence/incidence, morbidity, mortality, and burden (including financial) resulting from the problem.	☑ Yes<br>☐ No<br>☐ Unclear	1-3	
**Aim(s) of the guideline and specific objectives**				
6	Describe the aim(s) of the guideline and specific objectives, such as improvements in health indicators (e.g., mortality and	☑ Yes<br>☐ No	3	

	disease prevalence), quality of life, or cost savings.	☐ Unclear		
**Target populations**				
7a	Describe the primary population(s) that is affected by the recommendation(s) in the guideline.	☑ Yes<br>☐ No<br>☐ Unclear	2-3	
7b	Describe any subgroups that are given special consideration in the guideline.	☐ Yes<br>☑ No<br>☐ Unclear	NA	
**End users and settings**				
8a	Describe the intended primary users of the guideline (such as primary care providers, clinical specialists, public health practitioners, program managers, and policymakers) and other potential users of the guideline.	☑ Yes<br>☐ No<br>☐ Unclear	2-3, 6	
8b	Describe the setting(s) for which the guideline is intended, such as primary care, low- and middle-income countries, or inpatient facilities.	☐ Yes<br>☑ No<br>☐ Unclear	NA	
**Guideline development groups**				
9a	Describe how all contributors to the guideline development were selected and their roles and responsibilities (e.g., steering group, guideline	☐ Yes<br>☑ No	4	

	panel, external reviewer, systematic review team, and methodologists).	☐ Unclear		
9b	List all individuals involved in developing the guideline, including their title, role(s) and institutional affiliation(s).	☐ Yes ☑ No ☐ Unclear	NA	
**Evidence**				
**Health care questions**				
10a	State the key questions that were the basis for the recommendations in PICO (population, intervention, comparator, and outcome) or other format as appropriate.	☐ Yes ☑ No ☐ Unclear	NA	
10b	Indicate how the outcomes were selected and sorted.	☐ Yes ☑ No ☐ Unclear	NA	
**Systematic reviews**				
11a	Indicate whether the guideline is based on new systematic reviews done specifically for this guideline or whether existing systematic reviews were used.	☑ Yes ☐ No ☐ Unclear	4	
11b	If the guideline developers used existing systematic reviews, reference these and describe how those reviews	☐ Yes ☑ No	NA	

	were identified and assessed (provide the search strategies and the selection criteria, and describe how the risk of bias was evaluated) and whether they were updated.	☐ Unclear		
**Assessment of the certainty of the body of evidence**				
12	Describe the approach used to assess the certainty of the body of evidence.	☑ Yes<br>☐ No<br>☐ Unclear	5	
**Recommendations**				
**Recommendations**				
13a	Provide clear, precise, and actionable recommendations.	☐ Yes<br>☑ No<br>☐ Unclear	NA	
13b	Present separate recommendations for important subgroups if the evidence suggests that there are important differences in factors influencing recommendations, particularly the balance of benefits and harms across subgroups.	☐ Yes<br>☑ No<br>☐ Unclear	NA	
13c	Indicate the strength of recommendations and the certainty of the supporting evidence.	☐ Yes<br>☑ No	NA	

		☐ Unclear		
**Rationale/explanation for recommendations**				
14a	Describe whether values and preferences of the target population(s) were considered in the formulation of each recommendation. If yes, describe the approaches and methods used to elicit or identify these values and preferences. If values and preferences were not considered, provide an explanation.	☐ Yes<br>☑ No<br>☐ Unclear	NA	
14b	Describe whether cost and resource implications were considered in the formulation of recommendations. If yes, describe the specific approaches and methods used (such as cost-effectiveness analysis) and summarize the results. If resource issues were not considered, provide an explanation.	☐ Yes<br>☑ No<br>☐ Unclear	NA	
14c	Describe other factors taken into consideration when formulating the recommendations, such as equity, feasibility and acceptability.	☐ Yes<br>☑ No<br>☐ Unclear	NA	
**Evidence to decision processes**				
15	Describe the processes and approaches used by the guideline development group to make decisions, particularly the formulation of	☑ Yes<br>☐ No	3-6	

	recommendations (such as how consensus was defined and achieved and whether voting was used).	☐ Unclear		
**Review and quality assurance**				
**External review**				
16	Indicate whether the draft guideline underwent independent review and, if so, how this was executed and the comments considered and addressed.	☑ Yes<br>☐ No<br>☐ Unclear	5-6	
**Quality assurance**				
17	Indicate whether the guideline was subjected to a quality assurance process. If yes, describe the process.	☑ Yes<br>☐ No<br>☐ Unclear	3-11	
**Funding, declaration and management of interests**				
**Funding source(s) and role(s) of the funder**				
18a	Describe the specific sources of funding for all stages of guideline development.	☑ Yes<br>☐ No<br>☐ Unclear	6	
18b	Describe the role of funder(s) in the different stages of guideline development and in the dissemination and implementation of the recommendations.	☑ Yes<br>☐ No<br>☐ Unclear	6	

**Declaration and management of interest**				
19a	Describe what types of conflicts (financial and nonfinancial) were relevant to guideline development.	☑ Yes<br>☐ No<br>☐ Unclear	6-7	
19b	Describe how conflicts of interest were evaluated and managed and how users of the guideline can access the declarations.	☑ Yes<br>☐ No<br>☐ Unclear	6-7	
**Other information**				
**Access**				
20	Describe where the guideline, its appendices, and other related documents can be accessed.	☑ Yes<br>☐ No<br>☐ Unclear	7	
**Suggestions for further research**				
21	Describe the gaps in the evidence and/or provide suggestions for future research.	☑ Yes<br>☐ No<br>☐ Unclear	6	
**Limitations of the guideline**				
22	Describe any limitations in the guideline development process (such as the development groups were not multidisciplinary or	☐ Yes<br>☑ No	6	

	patients' values and preferences were not sought), and indicate how these limitations might have affected the validity of the recommendations.	☐ Unclear		

Reference:
Chen Y, Yang K, Marušić A, et al. A Reporting Tool for Practice Guidelines in Health Care: The RIGHT Statement[J]. Annals of Internal Medicine, 2017, 166(2):128-132. https://doi.org/10.7326/M16-1565
Official Website: http://www.right-statement.org/
